# Solitary fibrous tumor of the submandibular region

**DOI:** 10.3892/ol.2014.2785

**Published:** 2014-12-09

**Authors:** WANG SHI, ZHENG WEI

**Affiliations:** 1 Department of Stomatology, The Third Central Hospital, Tianjin 300170, P.R. China; 2 Department of Orthodontics, Tianjin Stomatological Hospital, Nankai University, Tianjin 300041, P.R. China

**Keywords:** solitary fibrous tumor, submandibular region, immunohistochemistry

## Abstract

Solitary fibrous tumors (SFTs) are a rare type of neoplasm that originate from the pleura. Although SFTs may occur in a variety of extrathoracic regions, they are considered to be rare in the submandibular region. The current study presents the case of a 39-year-old female with a 3×4-cm, fibro-elastic, movable, painless nodule in the right side of the submandibular region. The patient exhibited no other clinical manifestations in the head and neck region. A computed tomography scan demonstrated the presence of a well-defined, slightly low-density nodular shadow measuring 2.6×3.3×3.8 cm, in proximity to the right submandibular gland, with mild contrast enhancement and no association with the adjacent lymph nodes. The lesion was surgically excised, and following histopathological and immunohistochemical analysis, immunohistochemical staining determined that the lesion was positive for cluster of differentiation (CD)34, CD99 and vimentin, and negative for desmin, CD31 and S-100; therefore, a diagnosis of an SFT was determined. The patient has so far been followed up for 22 months, with no signs of recurrence or metastases. The present study also discusses the clinical, histopathological and immunohistochemical features, treatment strategies and potential clinical outcomes of SFTs. The study proposes that, although rare, SFTs of the submandibular region should be included in the differential diagnosis of soft-tissue tumors in the submandibular region.

## Introduction

Solitary fibrous tumors (SFTs) are a rare type of spindle cell neoplasm that typically arise in the pleura. SFTs were initially differentiated from mesothelioma in a study by Klemperer and Coleman (1) in 1931. The tumors generally affect middle-aged adults, however, they have also been identified in young patients. Furthermore, SFT affects males and females at the same frequency (2,3). SFT has been described in almost every organ in the human body, however, presentation in the submandibular region is rare, with only three previously reported cases (4–6). The histogenesis of SFT has previously been debated, however, recent studies have indicated that it is of mesenchymal origin (7–9). A definitive diagnosis of SFT may be determined by pathological examination, including immunohistochemical and molecular techniques, particularly staining for cluster of differentiation (CD)34, which is considered to be a marker of SFT (10). In addition, the majority of cases of SFT are diffusely positive for CD99 and B-cell lymphoma 2 (Bcl-2) (11). The current study presents a patient who underwent surgery for a submandibular lesion, which was determined to be SFT following pathological analysis. The present study reports a case of SFT affecting the submandibular region and reviews the relevant literature on previously reported cases. Furthermore, clinical, histopathological, immunohistochemical and treatment strategy aspects of the tumor are discussed. Written informed consent was obtained from the patient.

## Case report

**Figure 1:**
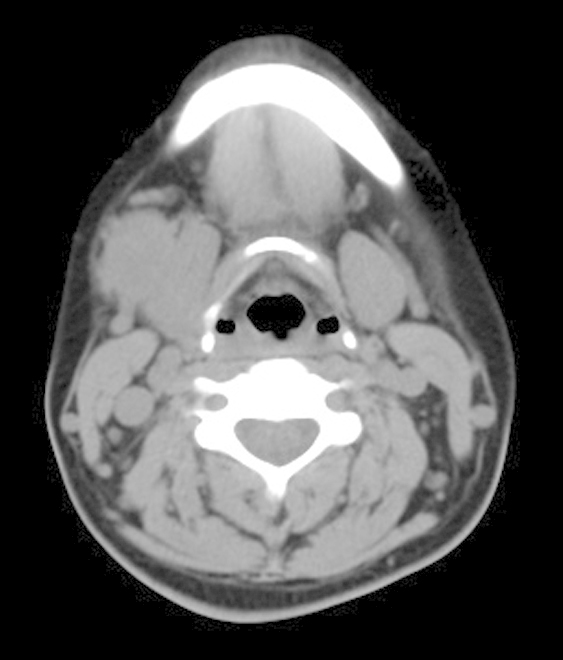
Computed tomography scan of a soft-tissue mass located adjacent to the right submandibular gland.

In April 2012, a 39-year-old female patient presented to The Third Central Hospital (Tianjin, China) with a one-week history of a progressively enlarging, painless nodule in the right side of the submandibular region. The patient exhibited no other clinical manifestations in the head and neck region, and no symptoms, such as fever, dyspnea, odynophagia, night sweats or weight loss. No significant personal or family medical history was noted and the patient had no history of tobacco or alcohol use. A physical examination revealed a 3×4-cm, fibro-elastic, movable, non-tender mass in the right submandibular triangle, however, no cervical lymphadenopathy was identified and the laboratory data was unremarkable. A computed tomography scan identified the presence of a well-defined, marginally low-density nodular shadow measuring 2.6×3.3×3.8 cm, in proximity to the right submandibular gland, with mild contrast enhancement and no associated adjacent lymph nodes (Fig. 1). Extirpation of the tumor was performed under general anesthesia. During the surgical procedure, a well-demarcated mass was identified; the tumor was well-circumscribed and did not involve the submandibular gland, therefore, it could be removed easily. The post-operative recovery was uneventful. The surgical specimen was a 2.2×3.0×3.5-cm soft-tissue mass that was gray-white in color, oval-shaped and firm in consistency. Microscopically, the lesion consisted of spindle-shaped cells with scant cytoplasm accompanied by prominent hyalinized collagenous tissue, which displayed hemangiopericytomatous patterns. The cells did not display cytological atypia and no mitotic figures were detected. In addition, immunohistochemical staining was positive for CD34, CD99 and vimentin, and negative for desmin, CD31 and S-100 (Fig. 1); these histopathological and immunohistochemical findings were used to determine a diagnosis of an SFT. The patient has so far been followed up for 22 months, with no signs of recurrence or metastases.

## Discussion

SFTs are a rare type of neoplasm that develop from membranes, most commonly the pleura, but also the peritoneum and meninges (12). Additionally, SFTs have previously been reported to occur in the paranasal sinuses, larynx, thyroid, pelvis, sublingual gland, cervix, ovary, kidney, tongue and skin. SFT was initially referred to as localized fibrous mesothelioma, solitary fibrous mesothelioma or submesothelial fibroma, as it was previously considered to originate from mesothelial cells and submesothelial fibroblasts (13). Subsequent studies, utilizing tissue culture and immunohistochemistry, demonstrated that these neoplasms are actually of mesenchymal origin, which explained the increasing body of literature identifying SFT in extrathoracic primary locations (14). Furthermore, although SFT is considered to occur most commonly in the pleura, continued reports of SFT located in extrapleural sites have recently challenged this evidence (15). SFT has now been described in almost every organ in the human body, including regions of the head and neck, such as the nose and paranasal sinuses, nasopharynx, major salivary glands, larynx, thyroid gland, skin, deep soft tissue, oral cavity, parapharyngeal space and orbit (16).

SFT is most commonly a benign neoplasm that exhibits benign clinical behavior (17). However, malignancy may occur and is associated with the histological features of marked hypercellularity and pleomorphism, infiltrative borders, necrosis and greater than four mitoses per 10 high-power fields. Additionally, a large tumor size and neoplasms located in the pleura, mediastinum, abdomen, pelvis or retroperitoneum are associated with more aggressive tumor behavior. Metastasis in SFT is rare, however, metastases have been described in the lungs, bone and liver (18).

The differential diagnosis of submandibular region masses includes pleomorphic adenoma, adenoid cystic carcinoma, squamous cell carcinoma, adenocarcinomas, lymphomas, schwannoma and sarcomas (19).

SFTs are typically characterized by well-demarcated, solid lesions, with a gray-white color on cross-section, and a lack of necrosis or hemorrhage. Microscopically, SFTs contain irregular hypo- and hypercellular areas and demonstrate long spindle cell proliferation, the cytoplasm of which appears small with ill-defined borders. SFTs are difficult to diagnose based on clinical and histological findings alone, therefore, immunohistochemistry is an essential complimentary assay (20). SFTs characteristically exhibit strong staining for CD34, Bcl-2, CD99 and vimentin and are typically negative for cytokeratin, S-100, smooth muscle actin and desmin (18). Furthermore, SFTs are non-reactive for actin, CD117, epithelial membrane antigen, factor VIII and D2-40; however, 2–3% of cells demonstrate Ki-67 nuclear positivity, and 90–95 and 70–75% of cases exhibit CD34 and CD99 positivity, respectively (21). The strong CD34 immunoreactivity of SFTs is particularly important in differentiating the neoplasm from hemangiopericytoma, which does not stain as consistently or intensely for CD34 (22).

The preferred treatment strategy for SFTs is complete local surgical excision (23). The requirement for cervical lymph node dissection is not indicated. Certain studies have reported favorable results with post-operative chemotherapy and radiotherapy in cases of incomplete resection (24–27). Although SFTs are typically benign, close follow-up is recommended due to their unpredictable metastatic and recurrent behavior.

In conclusion, the present study reported a case of SFTs involving the submandibular region with clinicopathological features and CT findings. During surgery the tumor was well-circumscribed and did not involve the submandibular gland. SFTs are rare and should be considered as a possible diagnosis when tumors of unknown origin are identified in the submandibular region.
